# Systemic Treatment of Breast Cancer. 1st Central-Eastern European Professional Consensus Statement on Breast Cancer

**DOI:** 10.3389/pore.2022.1610383

**Published:** 2022-07-11

**Authors:** Gábor Rubovszky, Judit Kocsis, Katalin Boér, Nataliya Chilingirova, Magdolna Dank, Zsuzsanna Kahán, Dilyara Kaidarova, Erika Kövér, Bibiana Vertáková Krakovská, Károly Máhr, Bela Mriňáková, Béla Pikó, Ivana Božović-Spasojević, Zsolt Horváth

**Affiliations:** ^1^ Department of Clinical Pharmacology, National Institute of Oncology, Chest and Abdominal Tumours Chemotherapy “B”, Budapest, Hungary; ^2^ Center of Oncoradiology, Bács-Kiskun County Teaching Hospital, Kecskemét, Hungary; ^3^ Department of Oncology, Szent Margit Hospital, Budapest, Hungary; ^4^ Clinic Center of Excellence, Heart and Brain Hospital, Science and Research Institute, Medical University-Pleven, Pleven, Bulgaria; ^5^ Oncology Centre, Semmelweis University, Budapest, Hungary; ^6^ Oncotherapy Clinic, University of Szeged, Szeged, Hungary; ^7^ Kazakh Institute of Oncology and Radiology, Almaty, Kazakhstan; ^8^ Institute of Oncotherapy, Faculty of Medicine, University of Pécs, Pécs, Hungary; ^9^ 1st Department of Oncology, Faculty of Medicine, Comenius University, Bratislava, Slovakia; ^10^ Medical Oncology Department, St. Elisabeth Cancer Institute, Bratislava, Slovakia; ^11^ Department of Oncology, Szent Rafael Hospital of Zala County, Zalaegerszeg, Hungary; ^12^ County Oncology Centre, Pándy Kálmán Hospital of Békés County Council, Gyula, Hungary; ^13^ Institute for Oncology and Radiology of Serbia, Daily Chemotherapy Hospital, Belgrade, Serbia

**Keywords:** early breast cancer, locally advanced breast cancer, adjuvant treatment, neoadjuvant treatment, metastatic breast cancer, inflammatory breast cancer, guideline

## Abstract

This text is based on the recommendations accepted by the 4th Hungarian Consensus Conference on Breast Cancer, modified based on the international consultation and conference within the frames of the Central-Eastern European Academy of Oncology. The professional guideline primarily reflects the resolutions and recommendations of the current ESMO, NCCN and ABC5, as well as that of the St. Gallen Consensus Conference statements. The recommendations cover classical prognostic factors and certain multigene tests, which play an important role in therapeutic decision-making. From a didactic point of view, the text first addresses early and then locally advanced breast cancer, followed by locoregionally recurrent and metastatic breast cancer. Within these, we discuss each group according to the available therapeutic options. At the end of the recommendations, we summarize the criteria for treatment in certain rare clinical situations.

## Introduction

Since the 3rd Hungarian Breast Cancer Consensus Conference (1), new evidence based on clinical trial results has been published, which has justified updating the 2016 recommendation. In addition to classical prognostic factors, certain multigene tests, which should be incorporated into the recommendations, will play an important role in therapeutic decision-making.

This professional guideline primarily reflects the positions and recommendations of ESMO (2), NCCN (3), ABC5 (4), and the St. Gallen Consensus Conference (5) (other sources are indicated in the appropriate section). From a didactic point of view, the text first addresses early and then locally advanced breast cancer, followed by locoregionally recurrent and metastatic breast cancer. Within this structure, we discuss each group according to the available therapeutic options. At the end of the recommendations, we summarize the criteria for treatment in certain rare clinical situations and propose the use of new protocols. Our recommendations basicly use the ESMO evidence and recommendation categories (and other, e.g., NCCN categories are indicated separately).

To achieve acceptable therapeutic results, it is important to treat patients with breast cancer in specialized institutions or departments where enough early-stage breast cancer patients are encountered each year (6). The European recommendation sets this number at 150 new cases per year (III.A).

Application of multidisciplinary principles is essential (7). Therapeutic decisions should be based on tumour board decision, involving representatives of the relevant specialties such as surgeons, pathologists, radiotherapists, radiologists, medical oncologists (and in certain cases psychologists) as well as the patient, or her legal representative/guardian as substitute decision-maker, and the patient’s decision-making support system. It is advised to involve clinical geneticist and fertility preservation specialist, too (in certain countries it is mandatory). The patient must be provided with the appropriate information on which to base her/his decisions. Due to their predicament, patients only retain a fraction of the information provided to them, therefore it is important to communicate in an easy-to-understand format with repetitions, to answer any of the patient’s questions, and in addition to passing verbal information to provide the patient with supplementary information in written format and patient-centred websites (V.A).

Some recommendations in the professional guidelines are considered off-label treatments at the time of the completion of this text; these are indicated with a “§” symbol.

## Early Breast Cancer

The primary decision point of early breast cancers is whether the tumour contains an invasive component.

Adjuvant systemic therapy decision in early breast cancer is based on known prognostic and predictive factors and patient preferences. Within this framework, the most important clinical task, beyond the assessment of the extent of the disease or identification of certain molecular subgroups, is to include a weighing of the expected risks and benefits of a given therapy. From this standpoint it is essential to pinpoint the therapies to which the tumour is expected to react or resist. Therefore, one of the most important factors is to determine the hormone sensitivity of the tumour (see Table 1). A tumour is considered hormone-sensitive if the oestrogen and/or progesterone receptor (ER/PR)—hereinafter hormone receptor (HR)—content is at least 1% positive cells (8, 9), even though the success of endocrine treatment is questionable in the presence of values less than 10%. It is important to note that the ER-negative/PR positive phenotype is exceedingly rare; in most cases, it is caused by a laboratory error or a false negative ER reading or a false positive PR reading. Therefore, a repeat of the tests should be requested (10).

**TABLE 1:** Hormone sensitivity categories of early breast cancer (Ref to pathology chapter).

Hormone sensitivity	Allred score	Recommended treatment
Highly hormone-sensitive	Allred 6^a^-7-8	Endocrine therapy is recommended [alone or after chemotherapy in combination (±anti-HER2)]
Hormone resistant	ER- and PR-negative (Allred 0 and 2)	Endocrine therapy is ineffective, chemotherapy (±anti-HER2) is required
Uncertain hormone sensitivity	Allred 3-5	Primarily chemotherapy ± anti-HER2, followed by endocrine therapy

a Allred score of 6 may be due to: 1) 10% to 1/3 of cells shows strong staining; 2) between 1/2 to 2/3 of cells shows moderate staining; 3) >2/3 of cells shows weak staining. See: Pathological diagnosis, work-up and reporting of breast cancer. Pathology recommendations from the 4th Consensus Conference on Breast Cancer.

Additional factors influencing the treatment:

• Tumour staging according to the TNM classification system,
• Pathological features beyond hormone-receptor status HER2 status, histological type, proliferation characteristics as grade, mitotic activity index (MAI), and Ki67,^1^
• Patient characteristics: biological age, general condition (performance status), comorbidities, organ reserves, previous treatment and preferences of the patient, availability of the medication, and results of genetic tests (gene expression profiles scoring and germ line mutation such as BRCA 1/2 among others, as indicated) (V.A).

In the next section we describe the treatment of breast cancer based on staging derived from the TNM classification system. A more accurate prognostic classification of TNM is represented by the AJCC prognostic staging that also takes differentiation into account (eighth edition) (11), and this is the same staging that is referenced in the Pathology chapter of the Consensus document. In the following section, tumours are classified according to anatomical stages. Classification systems such as NPI (Nottingham Prognostic Index) or the PREDICT tool (https://breast.predict.nhs.uk/) provide important additional prognostic information to assist therapeutic decision-making.

### Non-Invasive Breast Cancer (Stage 0, Tis, N0 M0)

• According to the combined assessment of two randomized studies, after breast-conserving surgery and radiation therapy (if any), 5 years of adjuvant tamoxifen therapy (20 mg/day) administered for hormone-sensitive tumours—independently from menopausal status - can decrease the incidence of invasive and non-invasive local recurrences as well as the incidence of secondary (contralateral) breast tumours (I.A).
• Compared to tamoxifen, anastrozole further decreased the incidence of breast cancer events, but no benefit was shown in terms of disease-free or overall survival (NRG Oncology/NSABP B-35 study) (12). With emphasis that AI are exclusively for postmenopausal patients, indication for aromatase inhibitor (AI) is like that of tamoxifen (I.B). Beyond menopausal status a different safety profile should be considered when choosing this medicinal product.
• If mastectomy is performed, the aim of postoperative treatment is to decrease the risk of a contralateral breast cancer, and therefore no additional systemic treatment is indicated following double mastectomy. When recommending adjuvant endocrine therapy (ET) it should be considered that—although the likelihood is small—clinically significant complications (such as endometrial cancer, thromboembolism, osteoporosis, and cardiovascular complications) may develop, and this treatment has no confirmed effect on survival.
• The prognosis of the disease is particularly good. At the 20 years mark the likelihood of an invasive cancer is 6% in the affected breast (breast-conserving surgery) and the contralateral breast alike, and tumour-specific mortality is 3%. The risk is higher for young (under 35 years old) patients if their tumour is poorly differentiated (grade 3, “high grade”) or oestrogen receptor negative (13).

#### Recommendation

• Chemotherapy and anti-HER2 therapy are not indicated.
• Aromatase inhibitor (anastrozole) and tamoxifen are both suitable as endocrine therapy (tamoxifen is preferable, AIs are not available in all countries.) Menopausal status should be considered.
• For radiotherapy, please see radiotherapy guideline.

### Early-Stage Invasive Breast Cancer (Stage I–II-IIIA [<N2]), or Potentially Resectable Stage IIIB

#### Criteria for Choosing Neoadjuvant and Adjuvant Systemic Treatments

• Decision-making on the use of perioperative (neoadjuvant or adjuvant) treatments has three steps.
  ○ The first step is the assessment of prognosis (See Supplementary Appendix S1).
  ○ The second step is assessment of predictive factors which will guide treatment choice. These two important factors will define the expected relative and absolute benefits of the treatment.
  ○ The third step is to consider potential short- and long-term adverse events as well as the patient’s characteristics and preferences into account (V.A).
• The prognosis of the tumour is defined mainly by its extent and biological characteristics. The prognostic factors of early invasive breast cancer include primary tumour size (T), nodal status (N), histological grade (G), proliferation rate (e.g. Ki67/MAI), HR and HER2 status, peritumoral vascular invasion, and recently, specific gene expression profile tests (genomic profiles). The assessment of prognosis is helped by the Nottingham Prognostic Index (14-17), tools analysing certain databases (PREDICT tool) (14, 18-21), and tumour genetic tests (see below).
• The expected efficacy of systemic treatment is indicated by the biological characteristics of the tumour (predictive factors). Recommendation of certain treatments should be based on treatment-oriented classification (Table 2) defined by the endocrine sensitivity (Table 1), HER2 status and proliferation characteristics of the tumour. Prognostic and predictive factors serve as a basis for deciding whether the therapeutic benefit of a given systemic treatment modality outweighs the risks due to its potential side-effects. As an example, for a patient with a good prognosis, clinically insignificant therapeutic gains can be expected from systemic treatment (primarily chemotherapy).
• Endocrine therapy is justified for all HR-positive conditions (ER and/or PR expression ≥1%) (I.A). In some countries adjuvant hormone treatment is recommended for all patients with HR-positive tumours without exceptions. Omission of endocrine therapy may be considered in other countries when the prognosis is remarkably good (the rate of long-term relapse is below 5%) (V.E).
• Chemotherapy may be omitted for a large proportion of HR-positive tumours, and its application is indicated primarily in more extensive and poorly differentiated tumours. Its use is usually justified in HR-negative cancers.
• Chemotherapy and anti-HER2 therapy are recommended for most HER2-positive cancers.
• Concomitant treatments:
  ○ Concomitant administration of chemotherapy and endocrine therapy is not recommended. The only exception is the administration of GnRH analogues to preserve fertility.^§^
  ○ Anti-HER2 therapy can be given concomitantly with taxane chemotherapy but not with anthracyclines (I.a) due to increased risk of cardiotoxicity. Also, it can be combined with radiation therapy^§^ and endocrine therapy; radiation therapy and endocrine therapy can also be administered concomitantly. Tamoxifen may exacerbate irradiation-induced pulmonary fibrosis, it could be considered for parallel application.
  ○ When adjuvant chemotherapy is indicated, it precedes radiation therapy.
• In recent years the classification of luminal A-like, luminal B-like, HER2-positive, and triple-negative has become the preferred method of decision-making in determining therapy. This classification derives from gene-expression profile results, where the tumours were grouped as luminal A, luminal B, HER2-enriched (HER2-E), basal-like and normal-like (normal-like is not considered a true, intrinsic subtype, as it originates from a tumour sample that includes a significant amount of normal breast tissue, thus representing a mix of normal tissue and tumour cells) (Table 2). Although multi-parametric genomic tests that serve as a basis for the classification are not widely available, immunohistochemical (IHC) tests can provide an approximation of the genome-based classes.^2^

**TABLE 2:** Surrogate definitions of the intrinsic subtypes of breast cancer (2).

Intrinsic subtype	Clinicopathological surrogate definitions
Luminal A “luminal A-like”	- ER-positive
	- HER2-negative
	- Ki67 low
	- PR elevated
	- Low-risk molecular signature (if available)
Luminal B “luminal B-like (HER2-negative)”	- ER-positive
	- HER2-negative
	- and - Ki67 high^a^ and/or
	- PR low and/or
	- High-risk molecular signature (if available)
“Luminal B-like (HER2-positive)”	- ER-positive
	- HER2-positive
	- Any Ki67^b^
	- Any PR
HER2 “HER2-positive (non-luminal)”	- HER2-positive
	- ER and PR missing
“Triple-negative”^c^	- ER and PR missing
	- HER2-negative^c^

Based on the recommendations of the 2013 St. Gallen Consensus Conference (162).

a Ki67 values should be evaluated based on local laboratory values: for example, if the median Ki67 value in the laboratory is 20% in HR-positive disease, then values of 30% or above are to be read as high, and values equal to or less than 10% should be read as unequivocally low. (However, since the median Ki67 values of each group determined by the laboratory are usually not known, the recommendation is not entirely reliable, and the values should be taken as general guidelines.)

b The recommended cut-off value is 20%; quality control programs are essential tools for laboratories for the evaluation of reports.

c There is an 80% overlap between the “triple-negative” and intrinsic “basal” subtype.

Additional factors that must be taken into account during the therapeutic decision include potential short-term and long-term side-effects (e.g., chronic alopecia, neuropathy, cardiac and vascular toxicity, second malignancy, infertility, and classical endocrine side-effects) as well as the biological age, general condition, comorbidities, and preferences of the patient.

• Adjuvant treatment should be started between 3–6 weeks after surgery but no later than the 12th week, because its efficacy declines significantly after this time point (I.A). Neoadjuvant therapy should be started after determining the diagnosis (based on the result of the core biopsy) and preferably within 4 weeks after mammography. It is preferable to have the staging results in hand before starting treatment, but a delay in obtaining these results should not delay the start of the treatment (V.A).

The treatment-oriented grouping recommended by current treatment guidelines differentiates four groups based on HR status, HER2 status and proliferation. The efficacy of the various treatment modalities is different in each subgroup (Table 2).

#### The Role of Gene Expression Assays and Other Molecular Diagnostic Tests in Determining the Choice of Adjuvant Chemotherapy and Endocrine Therapy

In addition to prognostic factors used to estimate risk of relapse and survival, molecular genetic tests (Oncotype DX^®^, MammaPrint^®^, PAM50 ROR^®^, Breast Cancer Index^®^, and EndoPredict^®^) add additional prognostic and possibly predictive information.

The available tests provide a variety of results. In general, clinical studies have been carried out in patients with stage pT1-2 and pN0 as well pN1 disease.

• The independent prognostic value of these tests is accepted and in the case of two tests (OncotypeDX^®^ and Mammaprint^®^) it is also supported by robust evidence (I.A).
• Regarding chemotherapy sensitivity (predictive value) OncotypeDX^®^ is the only test supported by evidence at present (I.A).

#### Recommendation (On the Application of Chemotherapy)

• Theoretically, if we want to make the best decision on the application of CT, all patients should be tested with Oncotype DX^®^.
• The OncotypeDX^®^ test is recommended in the case of ER-positive, HER2-negative, primarily stage pT1c-pT2 N0-N1mi M0 patients with “moderate risk” (3.4–5.4 based on NPI) early breast cancer patients, if the available tests results and other criteria do not allow the oncology team to define a clear therapeutic plan and the patient accepts chemotherapy as long as that is supported by the results of this test.
  ○Stage pT1bc-T2pN0 (HR-positive/HER2-negative) patients (22) were included in the TAILORx study.
      ▪ In general, an RS (recurrence score) below 26 indicated that the chemotherapy had no added value.
         ⋅ however, reduction in the number of distant metastases in response to chemotherapy was detected in the under 50 age group with RS 16–25, therefore chemotherapy should be considered for these patients (it provides 1.6% benefit in the RS 15–20 range and 6.5% benefit in the RS 21–25 range).
      ▪ In the range of RS 26–30 chemotherapy should be considered and rather recommended. For values above RS 30 the benefits of chemotherapy were clear (27% benefit) (23).
  ○ Based on the above data the simplified recommendation is as follows:
      ▪ RS 0–25: endocrine therapy (with additional ovarian suppression for patients younger than 50), but chemotherapy can be considered in individual cases.
      ▪ RS 26 and above: chemotherapy—the chemotherapy regimen is based on the patient’s general condition and preferences.
  ○Lymph node micrometastasis is classified as N1 by OncotypeDX^®^. Accordingly, in stage N1 (mic and 1–3) the following are recommended based on RS score.
      ▪ 0–25: in postmenopausal patients chemotherapy is not recommended.
      ▪0–25: in premenopausal patients chemotherapy should be considered, however, the therapeutic gain can be derived from ovarian suppression effect of chemotherapy. The absolute gain was more pronounced with RS 14–25.
      ▪ Above 25: chemotherapy is recommended.
• In cases of favourable histology (tubular, mucinous, papillary), the markedly favourable survival outlook means that chemotherapy is generally not required, and a multigene test is unlikely to be required.
• Limited data available from male patients (24) and in neoadjuvant situation, however the prognostic value of OncotypeDX is likely.

The availability of hormonal resistance biomarkers that can be confirmed by other molecular genetic methods has been steadily increasing (e.g., PIK3CA mutation, ESR1 and AKT mutations, HER2 and FGFR alterations, etc.). Currently their practical significance is mainly limited to advanced/metastatic disease, but thanks to the increasing availability of the promising liquid biopsy method all indicators point to their future use in the adjuvant and follow-up periods (25).

#### Criteria for Choosing Adjuvant Endocrine Therapy and Therapeutic Options

ET has an essential role in HR-positive and HER2-negative tumours. When choosing ET, menopausal status and risk should be considered (for relevant definitions see Supplementary Appendix S2). Since a variety of endocrine therapies with nearly identical effectiveness but partially different side-effect profiles are currently available, optimal strategies can be devised for each patient. The therapeutic plan should depend on the risk of relapse, molecular characteristics and histological subtypes of the tumour, the risk of contralateral breast cancer, age, especially menopausal status and patient’s preferences.

• In premenopausal women, ovarian ablation–if necessary—should be carried out using a reversible method (LHRH/GnRH analogue) due to the risks of prolonged oestrogen depletion [e.g., osteoporosis, (26)] and to preserve fertility for potential future childbearing (27) (II.B). Laparoscopic adnexectomy should be offered as an alternative, after properly informing the patient.
• Aromatase inhibitor (with LHRH/GnRH analogue if premenopausal) or tamoxifen (with or without LHRH/GnRH analogue if premenopausal) can be used as adjuvant endocrine therapy in both premenopausal and postmenopausal women, and their use should be adjusted according to the characteristics of the tumour and the side-effect profile of the administered medication (28, 29) (I.A). The menopausal level of oestrogen should be checked regularly during LHRH-analogue therapy.
• In HR-positive and HER2-positive cases, ET should be added to HER2 inhibitor therapy following chemotherapy (I.A).
• In HER2-positive and luminal B-like cases use of an aromatase inhibitor is preferable (with LHRH/GnRH analogue if premenopausal) (28, 29).
• Tamoxifen can be recommended as sole therapy for 5 years for low-risk, stage I hormone-sensitive breast cancer in both premenopausal and menopausal women (5).
• According to the SOFT study, ovarian ablation potentiates the effects of both tamoxifen and aromatase inhibitor in terms of distant metastasis-free survival (30, 31) (II.B).
• According to the SOFT and TEXT (27,29–34) studies, exemestan + LHRH/GnRH analogue (triptorelin)—compared to tamoxifen + LHRH/GnRH analogue (triptorelin)—improves disease-free survival and metastasis-free survival during premenopause, especially in high-risk patients. The greatest benefit can be expected in the high-risk patient group (<35 years of age, high grade/high Ki67 value, or positive lymph node status); this therapeutic benefit was especially compelling in patients who had already received chemotherapy (31) (I. A).
• Tamoxifen administered for 10 years to high-risk (primarily lymph node positive) premenopausal women provided a survival benefit compared to 5-year treatment (32, 33) (I.A). 5 years of AI therapy following 5 years of tamoxifen therapy also decreases disease-free and overall survival in lymph node-positive disease. The currently recommended period of aromatase inhibitor administration during extended endocrine therapy is 5 years, but studies in progress are evaluating this therapy beyond the 5 year period (32, 34, 35). According to a recent meta-analysis (36), compared to tamoxifen therapy or tamoxifen-aromatase inhibitor switch treatment strategies, extended aromatase inhibitor therapy provided significant advantage in terms of relapse-free survival (RFS) however did not reach a significant level in patients who were previously treated with AI monotherapy. The benefit increased with lymph node positivity, but there was also a significant increase in the number of events affecting the bones. Required check-ups before and during aromatase inhibitor therapy include routine bone density measurements every 2 years, or more frequently if the patient has osteoporosis.

#### Adjuvant ET Recommendations—Premenopause

• Tamoxifen for 5 years.
• Tamoxifen for 10 years.
• Aromatase inhibitor + LHRH analogue for 5 years ± ([extended] tamoxifen 5 years).
• Tamoxifen for 5 years + LHRH analogue (for 2–5 years) (± [extended] tamoxifen for 5 years).
• Tamoxifen (±LHRH analogue) for 5 years, and once patient is in stable menopause 5 years of aromatase inhibitor (however, menopause status must be confirmed).

#### Adjuvant ET Recommendations—Postmenopause

• Tamoxifen for 5 years if patient is low risk (stage I).
• Aromatase inhibitor for 5 years.
• Aromatase inhibitor and tamoxifen in any order (switch regimen)—2–3 years/3–2 years.
• The extended ET should be followed by tamoxifen or aromatase inhibitor according to prior adjuvant ET and side-effect profile.

During perimenopause, aromatase inhibitor therapy can induce stimulation of the ovaries, therefore hormone tests (repeated FSH and oestradiol tests are recommended) [I.a]) to increase the safety of the chosen therapy for women aged <60 years in menopause (see also the criteria for menopause, Supplementary Appendix S2). When endocrine therapy is started close to the anticipated time of menopause but still in premenopause—and the patient’s hormone tests later confirm postmenopause—the patient could be switched from tamoxifen to aromatase inhibitor therapy without the addition of LHRH analogue.

For HR-positive and HER2-positive cancers the standard therapy is chemotherapy followed by endocrine therapy plus a total of 1 year of trastuzumab treatment (I.A). If the patient is high-risk, taxane based chemotherapy is combined with dual anti-HER2 blockade (trastuzumab + pertuzumab if available), followed by endocrine therapy along with dual anti-HER2 blockade up to 1 year (see below). Consider extended neratinib therapy for one additional year in high-risk tumors, if available (also see later).

#### Criteria for Choosing Adjuvant Chemotherapy

The requirement for adjuvant treatment is based on the risk of relapse. Clear indications for cytotoxic chemotherapy include the following parameters that indicate high risk:

• Basal type/triple-negative or HER2-positive breast cancer (larger than 10 mm, at least pT1c) and/or pN1 (1–3 metastatic lymph nodes). Rare exceptions include such histological subtypes as secretory or adenoid cystic carcinoma (few available data, chemotherapy only in case of N+).
• High-risk luminal HER2-negative tumours (e.g., result of multigene test) (I.A).
• N2-N3 lymph node status (4 or more metastatic lymph nodes).

In HR-positive and HER2-negative disease other indications:

• G3; intermediate/high proliferation, high Ki67.
• Low HR content (less than 10% of tumour cells show positivity).
• pN1 status.
• Lymphovascular invasion.
• Large tumour mass (pT3-4) (based on the assumption that chemosensitive or endocrine resistant clones are present due to intratumoural heterogeneity).
• Age less than 35 years should not be the exclusive reason to give chemotherapy if other intermediate or high-risk factors are not present. In grey zone, multigene tests (if available) can be used to assist in determining the proper adjuvant therapy.
• Chemotherapy is primarily recommended for triple-negative (TN), HER2-positive and luminal B-like HER2-negative type tumours (I.A). The absolute benefit of chemotherapy is more pronounced in case of ER-negative tumours.
• The choice of chemotherapy depends on the expected efficacy but also depends on long-term toxicity of the chosen treatment, the biological age, general condition, comorbidities, and preferences of the patient (2).
• Most luminal A-like tumours do not require chemotherapy; the exception are cases with large tumour mass or extensive lymph node involvement (pT3-4 or pN2-3). According RxPONDER trial results in selected cases with pT3pN0-1 tumor chemotherapy may be avoided with RS (OncotypeDX^®^) (37, 38).
• Chemotherapy can be administered when the indication is uncertain (with all clinical and pathological factors known), and the result of the gene expression test, for example OncotypeDX^®^ are indicating intermediate or high risk for relapse. This was discussed above in detail.

#### Recommendation

• The standard chemotherapy treatments include anthracycline and/or taxane preferably as sequential therapy.
  ○ The most commonly used anthracycline-containing treatments are doxorubicin/adriamycin-cyclophosphamide (AC) and epirubicin-cyclophosphamide (EC) for four cycles (I.A) (3).
  ○ The most used taxane based chemotherapy in sequence with anthracyclines are mono-docetaxel for four cycles or mono-paclitaxel given once weekly for 12 weeks (3).
  ○ 5FU-containing triple drug combinations (FAC/FEC) should no longer be used routinely.
  ○ Taxane-based treatments (without anthracycline), such as docetaxel/cyclophosphamide (TC) (39), can be alternatives to anthracycline based-therapies. According to the US Oncology Trial 9735, in non-selected stage I-IIIB patients the docetaxel-cyclophosphamide combination is significantly more effective in terms of both DFS and OS than the doxorubicin-cyclophosphamide combination.
  ○ Treatments without anthracycline can be used in the presence of significant risk of cardiac complications (I.A).
  ○ The efficacy of the 6×CMF protocol is identical to that of 4×AC/EC, but its toxicity is greater (II.B)(40). The use of anthracyclines is preferred to treatment with the CMF regimen, because they are significantly more effective both in terms of relapse and survival given in the same number of cycles (41) (I.A).
• The inclusion of taxanes resulted in a moderate increase in therapeutic efficacy independently of age, lymph node status, grade, and receptor status (I.A). Overall, anthracycline- and taxane-based chemotherapy protocols decrease breast cancer mortality by a third.
• The sequential administration of taxanes and anthracycline is more beneficial and less toxic (due to reduced cardiotoxicity) than concomitant administration (I.A).
  ○ The recommended dosing frequency is once a week for paclitaxel and once every 3 weeks for docetaxel.
  ○ According to a randomized trial, the taxane/anthracycline order may be more effective than the usual anthracycline/taxane order, although both are acceptable (42) (I.A).
• In the absence of significant prospective data, the routine treatment of TN and/or BRCA1/2 positive tumours with platinum-containing therapies—even though they seem highly effective—is not recommended.
• Dose-dense (dose-intensified) treatments (supported by the administration of G-CSF) are primarily recommended for tumours with high proliferation rates (I.A). Based on long-term analyses, dose-intensified treatments are more effective than treatments with conventional schedules.
  ○ One such therapy is the AC—P protocol applied every 2 weeks with filgrastim support (CALGB 9741 trial) (43).
• If there is a high risk of recurrence and/or axillary lymph node positivity is confirmed, then sequentially.
  ○ 4×AC—12× (weekly) paclitaxel (E1199 trial) (44).
  ○ Or concurrently (6×docetaxel + AC, “TAC”/TEC) dosage is also possible (BCIRG001 trial) (45); in the latter case filgrastim prevention is used due to rates of febrile neutropenia in excess of 20% (not recommended in all countries).
  ○ According to the NSABP B-38 trial (46) (TAC vs. AT vs. AC—T)—which only included N0-1 patients—the sequential AC—T arm produced significantly better results in terms of both DFS and OS than the other two arms; the efficacies of the other two arms were identical. This trial also confirmed that survival parameters were much better when chemotherapy-induced amenorrhoea developed than in the absence of this side-effect.
• Triple-negative disease also warrants the use of anthracycline and taxane.
• The indication of chemotherapy for patients above 70 years is determined on an individual basis, and the biological age, comorbidities, and preferences of the patient should be included in the decision-making process. Only limited data are available from clinical trials. Medications should be used at full dose if possible. A geriatric status assessment is recommended before the planned treatment is initiated.
• Concomitant administration of chemotherapy and endocrine therapy is not recommended, with the exception of GnRH analogues used to preserve ovarian function (47) (I.A).
• Anti-HER2 treatment can be routinely combined with non-anthracycline-containing chemotherapy, endocrine therapy, and radiation therapy.
• If both chemotherapy and radiation therapy are required, chemotherapy must precede radiation therapy (48, 49). The exception to this rule is:
  ○ Capecitabine treatment following adjuvant radiation therapy of patients with residual tumour after neoadjuvant chemotherapy (50) (II.A).
• The use of equipment for administering venous systemic treatment—e.g., port catheter, indwelling peripheral cannula—should be considered, and decided on an individual basis for each patient, taking into consideration both their benefits and potential complications.
• On the postoperative side, all interventions that increase lymph circulation are associated with an increased risk of lymphoedema. Therefore, necessary interventions (such as blood pressure measurements, blood draw, and infusions) should be primarily performed on the contralateral extremity (III.B).
• High-dose chemotherapy with stem cell transplantation is not recommended.

#### The Treatment of Early HER2-Positive Tumours

**FIGURE 1:**
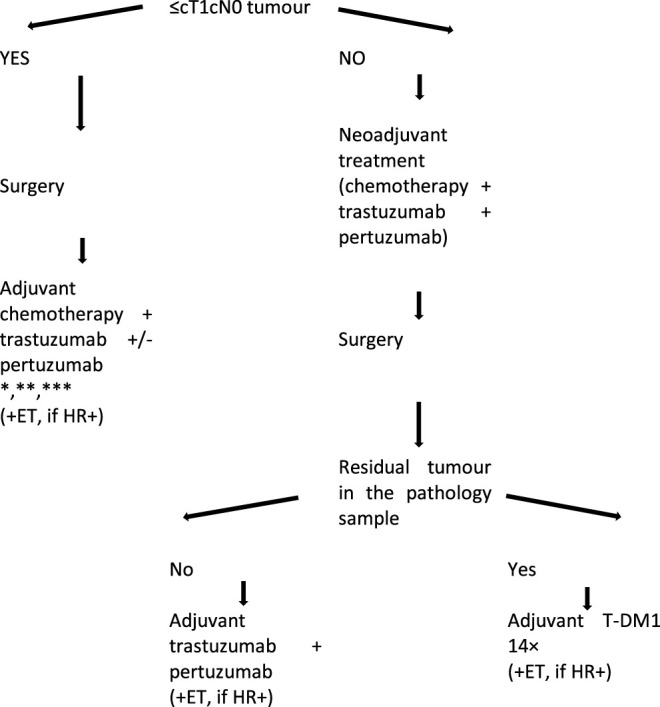
Treatment algorithm of HER2-positive early breast cancers. *Adjuvant administration of trastuzumab and pertuzumab for a total period of 1 year is recommended; this also includes the neoadjuvant cycles. **Adjuvant pertuzumab is recommended for high-risk patients (lymph node positivity). *** In pT1a cases neither chemotherapy nor anti-HER2 therapy is required, except for ET in the event of HR-positivity.

For early-stage (stage II-III) HER2-positive breast cancers the preferred treatment is neoadjuvant therapy containing HER2 targeted therapy, and the postoperative adjuvant treatment is determined by the degree of pathological response. However, for small and lymph node-negative tumours (stage I) primary surgery is also acceptable, and it is followed by adjuvant treatment containing anti-HER2 treatment (Figure 1).

Recommendations for neoadjuvant/primary systemic treatment of HER2-positive breast cancers:

For HER2-positive tumours the anti-HER2 therapy must be started as part of the primary systemic treatment.

• The standard base therapy is perioperative trastuzumab therapy for 1 year.
• Dual HER2 inhibition is recommended from stage II, using trastuzumab and pertuzumab (primarily for HR-negative cases) (51-54).
• HER2 inhibitor therapy should be started together/concurrently with taxane-containing neoadjuvant chemotherapy.
  ○ This means four cycles of (epi-) adriamycin-cyclophosphamide (AC/EC) treatment followed by weekly paclitaxel (12 cycles) OR docetaxel (4 cycles, every 3 weeks) AND trastuzumab ± pertuzumab treatment.
• Trastuzumab can be administered intravenously or subcutaneously.
• Concomitant administration of anti-HER2 therapy and anthracycline is not recommended due to increased cardiac risk.
• A non-anthracycline-containing protocol can be used in the presence of increased cardiac risk or in very young patients—e.g., Six cycles of TCH (docetaxel, cyclophosphamide, trastuzumab) or in the neoadjuvant setting PHTC (pertuzumab + trastuzumab + docetaxel + carboplatin) (TRYPHAENA and KRISTINE trials) (51, 52). Optimal use of the de-escalation regimens is not yet defined based on limited available data e.g., ADAPT-HR-/HER2+ (55), results of more studies (CompassHER2 (56) and DeCrescendo (NCT04675827)) should be awaited.
• All planned neoadjuvant chemotherapy (+ the associated concomitant anti-HER2) treatment cycles should be scheduled before surgery to increase the chance of pCR. The rest of the anti-HER2 treatments are administered as adjuvant therapy.
• Achieving pCR predicts a good prognosis with improved disease-free and overall survival and therefore should be the objective of treatment.

Recommendations for choosing adjuvant therapy following primary systemic chemotherapy + anti-HER2 treatment.

• If the patient achieves pathological complete response (pCR) in response to neoadjuvant therapy, continuation of trastuzumab + pertuzumab or trastuzumab monotherapy is recommended as adjuvant treatment, for a total of 1 year (including the neoadjuvant cycles). Based on the results of the APHINITY trial (57) the benefit of dual inhibition was primarily detected in lymph node positive patients (II.B). If there is residual invasive disease in the breast and/or the axilla, then surgery should be followed by the administration of 14 cycles of ado-trastuzumab emtansine (T-DM1) if available, because—based on the data of the KATHERINE trial (58)—significant and clinically relevant increase in disease-free survival can be achieved compared to adjuvant trastuzumab (II.B). If the T-DM1 has to be interrupted, e.g. due to toxicity, then the adjuvant treatment should be completed with trastuzumab (±pertuzumab) until the 1 year time point (Figure 1). In this trial most patients were administered radiation therapy and endocrine therapy concomitantly with postoperative trastuzumab or T-DM1 treatments if the tumour was HR-positive. The investigators could not detect any increase in treatment-associated toxicity associated with these concomitant treatments.
• According to the results from several neoadjuvant trials, the combination of trastuzumab and lapatinib^§^ (59) or trastuzumab and pertuzumab dual HER2 blockade^§^ (53, 54) can induce significant pCR even without chemotherapy, but the magnitude of the response lags behind that induced by combinations with chemotherapy. Currently there is no known biomarker that would selectively indicate patients who are suitable for treatment with biologic therapy only (without chemotherapy) therefore it is not recommended.

Recommendations for adjuvant only anti-HER2 therapy:

• The combination of chemotherapy and anti-HER2 therapy is required starting at pT1c status and lymph node positive disease (trastuzumab ± pertuzumab). It can also be recommended for the treatment of pN0 tumours measuring less than 1 cm in size (primarily pT1b, ER-negative) (IV.B).
  ○For very early, lymph node negative, low-risk disease with size below 5 mm (pT1a pN0) close observation can be considered, but administration of trastuzumab + paclitaxel is also supported (not recommended in all countries).
• Addition of pertuzumab to trastuzumab is primarily recommended for high-risk disease (lymph node positivity) as shown by the results of the APHINITY trial (57).
• Concomitant administration of anti-HER2 treatments and anthracycline therapy is not recommended.
• According to the principles of sequential treatment, four cycles of AC/EC, followed by 12 weeks of paclitaxel or four cycles of docetaxel + trastuzumab ± pertuzumab (HER2 targeted therapy started with taxane) treatment should be preferred.
• Chemotherapy is followed by trastuzumab ± pertuzumab therapy for a total of 1 year (I.A).
• In Stage I, non-anthracycline-containing, low toxicity TH (paclitaxel + trastuzumab 12×) treatment is also a viable option (60), especially in the presence of comorbidities (II.B).
• If the administration of anthracycline is to be avoided due to increased cardiac risk, then six cycles TCH(P) treatment (docetaxel + carboplatin + trastuzumab ± pertuzumab) or docetaxel + cyclophosphamide + trastuzumab can be recommended as non-anthracycline-containing options (II.B).
• Regular (or in the absence of problems, quarterly) cardiac follow-up (typically echocardiography) is required at the beginning of and during adjuvant trastuzumab ± pertuzumab treatment.
• After the completion of chemotherapy, endocrine therapy should be started concurrently with adjuvant anti-HER2 treatment (I.A).
• Adjuvant trastuzumab can be administered intravenously or, alternatively, in the fixed dose subcutaneous formulation, which is of equivalent efficacy and similar side-effect profile, and which does not require a loading dose. The latter method is more beneficial for the patient and is easier to administer (61-63). Subcutaneous trastuzumab can be given as monotherapy or as a combination with fix-dose subcutaneous pertuzumab (64).
• For high-risk, HR-positive, HER2-positive, lymph node-positive breast cancer, if available an extended adjuvant HER2 inhibitor option is the administration of neratinib for 1 year following trastuzumab-containing treatment^§^ (65). The benefits and toxicity profile of this treatment for patients receiving prior pertuzumab or adjuvant T-DM1 are not known, and therefore it is not recommended for this patient group.

## Preoperative/Neoadjuvant Systemic Therapy (Stages IIA-IIB-IIIA(N2)—Unresectable IIIB-IIIC)

When mastectomy is necessitated by large tumour size in cases of locally/regionally advanced (unresectable) and large resectable invasive tumours, primary (neoadjuvant) systemic treatment (PST) is recommended to reduce the extent of surgical intervention. The therapeutic response to PST has prognostic value and assists in choosing postoperative therapy.

All systemic therapeutic modalities (chemotherapy, endocrine and molecularly targeted therapy) used as adjuvant treatments can also be administered pre-operatively.

The advantage of PST is that systemic therapy can be started at the earliest time point and its efficacy can be measured based on tumour regression, and it can be used as an *in vivo* chemosensitivity test and thus serves as a starting point for treatment modification (if needed). Another advantage is that it decreases the risk of chemo resistance. By down staging the primary tumour or even axillary lymph node metastases, it can make originally unresectable tumours resectable, and can also moderate the extent of surgery and/or radiation therapy.

Pathological complete response (pCR) is a basic parameter indicating the efficacy of primary/neoadjuvant systemic therapy and predicting both the expected prognosis and survival. According to a widely used definition, pCR means that in response to neoadjuvant therapy an invasive tumour (or in some cases, *in situ* tumour) becomes undetectable in the surgically resected specimen at the site of the primary tumour and the lymph nodes (ypT0/ypTis ypN0). (For definitions, please see the chapter on pathology).

Neoadjuvant therapy is proven to be as effective as adjuvant therapy that is only administered after surgery.

• Once all the necessary pathology and staging reports are available to support clinical decision-making, neoadjuvant therapy must be initiated without delay. Ideally, there should be no more than 4–6 weeks between the first meeting with the patient and initiation of treatment (III.A).
• Neoadjuvant therapy is indicated at and above cT2 AND cN0 OR c/pN-positive status (including occult breast cancer). Neoadjuvant chemotherapy is recommended for all tumours larger than 2 cm in size where chemotherapy is otherwise indicated, and especially for TN and HER2-positive subtypes (I.B).
• During treatment, physical examination before each cycle and, if necessary, imaging check-ups of the patient at least after the second cycle are recommended, and placement of clip markers is required before treatment in cases in which there is a potential for breast-conserving surgery.
• If the primary unresectable tumour demonstrates regression in response to chemotherapy and/or chemotherapy plus biological therapy, completion of all the planned chemotherapy cycles before surgery is recommended (I.B).
• If the primary unresectable tumour does not show adequate remission, a change in the chemotherapy protocol or radiation therapy is recommended to achieve resectability.
• In case of progression or suspected progression, surgery should be performed if possible (except for inflammatory breast cancer, see below).
• When the breast cancer is resectable, the timing of chemotherapy (whether pre- or postoperative) does not influence the long-term disease course (II.C). For primary resectable cases, inadequate remission or even progression after the first 3 to 4 cycles indicates the need for surgery.
• If distant metastases are detected during neoadjuvant (primary systemic) treatment, the patient should be treated according to the recommendations given for metastatic breast cancer.
• Following successful surgery, the previously initiated treatment should be continued, with adjuvant systemic therapy as described below:
  ○ If the patient was not administered the entire course of preoperative chemotherapy, then completion of the previously (prior to surgery) successful combinations is recommended, or
  ○ If the patient finished the entire course of the planned neoadjuvant chemotherapy, then—outside of a clinical trial—further chemotherapy is contraindicated even in the absence of pCR. Exception is the TN and HER2+ patient group where in the case of residual tumour (not pCR) administration of 6–8 cycles of capecitabine in TNBC (50) (I.C) or in HER2-positive cases administration of T-DM1 (58) is justified (I.C).
• If an unresectable Stage III breast cancer cannot be made resectable even with neoadjuvant chemotherapy (±anti-HER2 ± endocrine therapy), further treatment must be defined on an individual basis (radiation therapy, chemotherapy, endocrine therapy) (V.C).
• Addition of a platinum derivative (usually carboplatin) to the usual treatment increases the likelihood of pCR in cases of TN breast cancer (I.C).
• Adjuvant administration of Olaparib for 1 year after completion of local treatment and (neo)adjuvant chemotherapy in high risk tumors significantly prolongs IDFS and DDFS in patients with germline BRCA1/2 mutations based on the results of OlympiA study (OS results are awaited) (66).
• Preferred adjuvant and neoadjuvant chemotherapy treatments for HER2-negative cases):
  ○Dose-dense AC (doxorubicin, cyclophosphamide) every 2 weeks, 4×, then paclitaxel every 2 weeks, 4×
  ○Dose-dense AC every 2 weeks, 4×, then weekly paclitaxel 12×
  ○TC every 21 days, 4–6× (docetaxel—cyclophosphamide) (recommended only as adjuvant therapy in Russia)
  ○Dose-dense AC/EC (epirubicin, cyclophosphamide) every 2 weeks
  ○ AC every 3 weeks, 4×, then paclitaxel weekly, 12×
  ○ AC every 21 days, 4×, then docetaxel every 21 days 4×
  ○ EC every 21 days, 8× (in selected cases; in some countries only 4–6 cycle are allowed by regulation).
  ○ TAC/TEC every 21 days, 6× (docetaxel, doxorubicin/epirubicin, cyclophosphamide) (not recommended in some countries; generally not preferable).
  ○ CMF every 28 days, 6× (in selected cases; recommended only as adjuvant therapy in certain countries).

### Primary Systemic Endocrine Therapy

• Neoadjuvant endocrine therapy can be used for tumours demonstrating strong HR expression. Since ER-positive/HER2-negative carcinomas, especially the lobular and luminal A-like subtype, are generally less sensitive to chemotherapy, endocrine therapy is expected to provide greater benefit.
• Sensitivity to the therapy can be predicted based on characteristics such as low grade, occasionally a special histological type (e.g. mucinous, tubular carcinoma), low Ki67 expression, high ER and PR expression, HER2-negativity, and slow progression. Similarly, a low OncotypeDX^®^ score is a predictor of good hormone sensitivity.
• Frequently, due to the general condition and age of the patient the physician is forced to administer primary ET for HR-positive tumours even in the absence of other signs of marked hormone sensitivity, and occasionally—unless followed by surgery—this primary ET remains the definitive therapy (V.C).
• For postmenopausal women the recommended length of primary ET before surgery is at least 6–8 months. If there is good response to the neoadjuvant ET, which is generally administered for 4–8 months or until maximal tumour response, then the therapy should be continued after surgery (I.A). If no regression can be detected after 2–4 months, a decision must be made whether to continue neoadjuvant therapy.
• When choosing neoadjuvant ET, the same rules must be followed as for adjuvant treatments.
• In premenopause, neoadjuvant endocrine therapy is not routinely recommended outside of clinical trials, although in selected luminal A-like tumours endocrine therapy (LHRH + AI) can be given as primary systemic therapy when the patient is not suitable for optimal surgery.
• Following surgery the proven, effective treatment is then continued as adjuvant therapy for 5–10 years. The postoperative treatment may be adjusted based on the histology of the surgical specimen, the extent of regression, the PEPI score (67), and phenotypical changes in the tumour.

## Primary Systemic Treatment of Inflammatory Breast Cancer (T4d)

From the clinical standpoint, the primary objective of the treatment of inflammatory breast cancer is to transform a primary unresectable tumour into a resectable one. Achieving maximal remission requires the use of the most effective treatment since minimal response or stable disease means that the tumour remains unresectable.

### Recommendation

• During evaluation/staging bilateral breast and lymph node assessments, imaging studies of the breast with MRI, PET/CT (or CT), and photographic documentation are recommended.
• The particulars of PST are identical to the medications and protocols used for the neoadjuvant therapy of non-inflammatory breast cancers (see above).
  ○ Sequential anthracycline-taxane combination is also the preferred treatment in this case and should be supplemented with trastuzumab and pertuzumab if the tumour is HER2-positive.
• The international expert committee primarily recommends a dose-dense chemotherapy regimen (AC, followed by paclitaxel, plus primary GCSF prophylaxis) (68, 69), but when taking into account cardiac toxicity the EC regimen is also considered to be acceptable.
• Multidisciplinary treatment of inflammatory breast cancer may include primary radiation therapy in addition to PST.
• Following successful PST, modified radical mastectomy, axillary dissection (I.B) and post-mastectomy irradiation (II) are recommended even for patients with complete response.
• Institution of adjuvant after-treatment based on prognostic and predictive factors is recommended, as described in the chapter on neoadjuvant therapy.

## Postoperative Systemic Treatment of Locally Recurrent, Resectable Breast Cancer

A local recurrence is predictive of a high risk of metastasis and/or additional local recurrences, and therefore administration of systemic therapy (chemotherapy and/or hormone therapy) and, if possible, radiation therapy should always be considered in such cases.

• Based on a small trial (the CALOR trial) (70) chemotherapy is only likely to provide a benefit if the tumour was HR-negative (the extent and biological properties of the primary tumour are not relevant in this case).
• The following factors must be considered when choosing a systemic treatment:
  ○ The biological characteristics of the resected tumour specimen (biopsy) (the receptor assays must be repeated!)
  ○ Previously used protocol(s) and administered doses
  ○ Time elapsed between the primary tumour and recurrence
  ○ General condition, comorbidities, organ reserves, and preferences of the patient (V.A)
• Notes:
  ○ Use of osteoclast inhibitors in early breast cancer is discussed later.
  ○ The use of CDK4/6-inhibitors are investigated also in (neo)adjuvant setting. No clear recommendation can be given, in the MonarchE trial abemaciclib combined with ET demonstrated a significant improvement in IDFS in patients with HR+, HER2-node-positive EBC at high risk of early recurrence (71, 72).
  ○ Adding immunotherapy (PD-1/PD-L1 inhibitor) to chemotherapy in triple-negative tumours is an issue. In the phase 2 GeparNuevo trial additional durvalumab improved long term outcome (iDFS, DDFS, OS) (73). In the phase 3 KEYNOTE-522 trial additional pembrolizumab improved EFS irrespective of PD-L1 status (74).

### Recommendation

• Hormone therapy and, if possible, radiation therapy should always be considered
• Chemotherapy is only likely to provide a benefit if the tumour was HR-negative

## Systemic Treatment of Locally Advanced (Unresectable) and Distant Metastatic Breast Cancer (Stage IV)

### Criteria for Choosing Systemic Treatments

• Metastatic breast cancer is usually incurable, but with carefully chosen treatments good response and maintenance of stable disease with minimal/acceptable side-effects can yield long-term survival. The objectives of palliative therapy are alleviation of symptoms, improvement of the patient’s quality of life, and the extension of life expectancy (V.A).
• Metastatic breast cancer is primarily treated with systemic therapy and/or radiation therapy, and surgery is performed only in a small fraction of cases with stable oligometastatic disease.
  ○ Palliative surgery and radiation therapy can be considered for brain metastases, meningeal-spinal cord compression, pleural, pericardial, biliary duct, or ureter obstruction, pathological or imminent pathological bone fractures, and localized painful bone or soft tissue metastases, while for liver metastases or cutaneous metastases on the chest wall, regional intra-arterial chemotherapy can be considered in individual cases and carefully selected patients^§^.
• Systemic treatment is chosen according to the following factors:
  ○ The biological behaviour of the tumour.
  ○ The extent of the tumour.
  ○ The general condition and biological age of the patient.
  ○ Comorbidities, possible drug interactions, previous treatments, disease-free interval, and patient preference.
• Chronological age (for elderly patients) or over-treatment (in the case of young patients) is not an acceptable reason to avoid treatment (I.E).
• The recommendation is to perform a biopsy of the metastasis or metastases whenever is approachable and possible to determine prognostic and predictive factors, primarily at the time of appearance of the first metastasis (I.B).^3^
  ○ Biopsy can be omitted if it has no therapeutic consequences, cannot be carried out due to the general condition of the patient, or is not feasible for technical reasons. If a biopsy cannot be performed, a liquid biopsy may be an alternative option if available (e.g., for PI3K mutation status).
  ○ It should be kept in mind that lesions assumed to be metastases may in fact hide a second primary tumour, which is another reason to support biopsy.
  ○ If the pathological characteristics of the primary tumour and the metastasis are different, there are no clear rules as to which lesion should guide treatment. In such cases the specimens should be compared and re-evaluated. The choice of treatment should be primarily based on the latest pathology report (V.B).
• The biological characteristics of the tumour are key determinants of palliative pharmacological therapy. Endocrine therapy is recommended for most HR-positive and HER2-negative tumours (see section on *Endocrine Therapy*). The exceptions are cases where visceral crisis is diagnosed and when hormonal resistance is suspected or proven. A visceral crisis is not the equivalent of visceral metastasis; rather, it is a major organ dysfunction characterized by rapid progression of symptoms and complaints, laboratory abnormalities, and the disease. Visceral crisis is present if the metastasis leads to the rapidly progressing decrease of organ functions (most frequently liver failure, respiratory failure, bone marrow failure, leptomeningeal infiltration etc.) (4). This is a situation where effective therapy is indicated in a narrow time frame, particularly because if the efficacy of the treatment is inadequate, further treatment is not feasible due to progression and the consequent worsening of the patient’s general condition. Apart from a few outliers, chemotherapy is the basis of treatment for HR-negative and HER2-positive (HR + or HR−) diseases. Chemotherapy should be supplemented with targeted therapy following biomarker studies.

### Endocrine Therapy for Metastatic Breast Cancer

• Endocrine-based therapy (ET) is the recommended primary treatment option for HR-positive and HER2-negative disease even in the presence of visceral metastases, except for visceral crisis or primary endocrine resistance (I.A). Primary endocrine resistance is present if the previous endocrine therapy/therapies was/were ineffective (see below).
• Molecular targeted agents (CDK4/6 inhibitors, everolimus and PIK3CA inhibitors) are parts of standard ET (if available); preferably as early-line treatments matched to the sequence and effects of prior therapies.
• Treatment is administered continuously until progression, and toxicity is usually not a limiting factor. Sooner or later hormone resistance can develop, necessitating a change in therapy, which is usually one of the next line of agents if the prior agent produces a good response.

The choice of endocrine treatment is influenced by prior therapies:

• The choice of first-line ET depends on the type and duration of adjuvant ET as well as the time elapsed since the completion of adjuvant ET.
• They may include AI, TAM, and fulvestrant, with the addition of ovarian ablation/suppression (medicinal or surgical) for pre- and perimenopausal women (I.A). In pre- and perimenopause, laparoscopic bilateral oophorectomy provides hormone depletion (as well as birth control) and helps avoiding tumour flare reaction caused by LHRH agonists (I.C)

#### Recommendations for endocrine therapies—in premenopause patients with HR+ mBC

• In addition to ovarian ablation/suppression with an appropriate agent (LHRH analogue or oophorectomy) the postmenopause algorithm should be followed with or without targeted therapy (I.A). In premenopausal status the basis of first-line ET is therefore ovarian ablation/suppression (I.A). For patients refusing ovarian ablation/suppression, tamoxifen monotherapy can be administered as optional ET although this method is less effective (I.D).
• Targeted molecular agents increase the efficacy of conventional ET and significantly impact on the overall survival of the patients. As metastatic first-line therapy, combination of CDK4/6 inhibitors with non-steroidal aromatase inhibitor (NSAI) significantly improved median PFS and OS in MONALEESA-7 trial (75, 76).

#### Recommendations for Endocrine Therapies—In Postmenopause Patients With HR+ mBC

As first-choice treatment for metastatic disease, combination of CDK4/6 inhibitors with NSAI significantly improved median PFS (24–25 months) in all clinical trials [PALOMA-2 (77), MONALEESA-2 (78, 79), MONARCH-3 (80)] with acceptable side-effects for (non-NSAI-resistant, see below) patients who had not received prior ET or progressed after previous adjuvant therapy. OS benefit was also proven in the “first-line” MONALEESA-2 trial. Therefore, this is the first-choice therapy recommended for postmenopausal patients (supplemented with ovarian ablation/suppression in pre- and perimenopause, NCCN category 1), and for men (supplemented with an LHRH agonist based on the MONALEESA-3 trial). In cases of NSAI resistance, this therapy should be combined with fulvestrant in the first line (PALOMA-3 (81, 82), MONALEESA-3 (83-85), MONARCH-2 (86, 87) trials). There is no data on continued treatment with CDK4/6 inhibitor after progression, therefore its use is not recommended beyond progression.

#### Recommended Combinations

• NSAI (anastrozole, letrozole) + CDK4/6 inhibitor (abemaciclib, palbociclib, ribociclib) (I.A).
• Fulvestrant + CDK4/6 inhibitor. The benefit of first-choice fulvestrant is confirmed for endocrine therapy naive patients with only bone metastasis (88) (II.B). For patients who were only given ET as first-line treatment, the combination of CDK4/6 inhibitor + fulvestrant resulted in a median PFS extension of 5–7.5 months (9.5–20.5 months) and improved quality of life [abemaciclib (89), palbociclib (90), ribociclib (85)]. This combination showed an OS benefit in the MONALEESA-3 trial (91, 92) (ribociclib) for postmenopausal patients, and in the MONARCH-2 trial (93) in both pre-/perimenopause and postmenopause (abemaciclib) (I.A).
• In the event of progression after combination NSAI + CDK4/6 inhibitor treatment, fulvestrant is considered standard therapy (94).
• Everolimus + exemestan:
  ○ Progressed during or within 12 months after completion of adjuvant treatment, OR.
  ○ For patients who progressed during or within 1 month after completion of treatment of advanced disease (91,95).
  ○ Supplemented with LHRH for male patients^§^.
  ○ In pre- or perimenopause, in addition to ovarian ablation/suppression since it significantly extends PFS but provides no OS benefit (I. B).
  ○ However, side-effects associated with combination treatment should be taken into consideration for this treatment (I.B). Tamoxifen (96) and fulvestrant (97) can also be combined with everolimus^§^ (II.B).
  ○ The benefit of everolimus administered after CDK4/6 inhibitor is currently unknown.
• Fulvestrant 500 mg + anastrozole (II.B) (98).
• Endocrine monotherapy:
  ○ NSAI (anastrozole, letrozole) (NCCN category 1).
  ○ SAI (steroid aromatase inactivator; exemestan).
  ○ Selective ER down-regulator SERD (fulvestrant 500 mg) (NCCN category 1).
  ○ Selective ER down-modulator SERM (tamoxifen).
• Abemaciclib as monotherapy—after prior ET and CT^§^ (99) (III.C).
• In case of known PIK3CA mutation (exon 9 or 20) after AI, (approved in Europe only after AI monotherapy) fulvestrant + alpelisib^§^ (100) (I.B)].
• As a third or subsequent choice, a treatment not previously used can be considered:
  ○ Tamoxifen
  ○ The choice of ET is influenced by prior treatment: if progression was detected during or <12 months after NSAI therapy used as adjuvant endocrine therapy, no good therapeutic effect can be expected (acquired resistance), and the next line fulvestrant should be chosen. Finally, in subsequent lines the following medications can be considered: tamoxifen or possibly exemestan.
  ○ Gestagens (megestrol acetate and medroxyprogesterone acetate).
  ○ Low-dose oestrogen (a few studies were published on its use after the development of endocrine resistance).
  ○ Insufficient evidence is available on re-challenge with certain previously used and effective agent, but it can be tried (IV.C).

Currently there are no known predictive biomarkers for the selection of patients for whom targeted therapies (CD4/6 inhibitors and mTOR inhibitor) would be beneficial and of the best choice among these therapies. Therefore, after considering the known side-effects, these therapies can be used for the treatment of all patients without exception if they are in line with the criteria described in the summary of product characteristics and visceral crisis is not present (I.E). There is no data available for the scenario of progression during CDK4/6 inhibitor treatment and successful switching to a different CDK4/6 inhibitor treatment, or whether switching ET would be beneficial clinically. Similarly, in the case of progression during everolimus therapy there is no evidence that everolimus would be effective when used in a different combination (NCCN category 1).

• Concomitant administration of chemotherapy and ET does not demonstrate any survival benefit, and therefore this combination is not recommended outside of a clinical trial (II.D).
• Chemotherapy followed by continuing treatment with maintenance ET is the logical next step in preserving the benefits of treatment, but data from randomized trials are not available at this point (II.B). Maintenance treatment after chemotherapy with ET plus CDK4/6 inhibitors is not recommended (ABC5).

### The Definition of Hormone Resistance (According to ABC5)

The choice of endocrine therapy is influenced by prior treatment: if progression was detected during or <12 months after NSAI therapy used as adjuvant endocrine therapy, no good therapeutic effect can be expected (acquired resistance), and the next line fulvestrant should be chosen (I.A).

#### Primary Endocrine Resistance

• Relapse during adjuvant ET (within the first 2 years).
• Progression developed during the first 6 months of first-line ET for metastatic disease.

#### Secondary Endocrine Resistance

• Relapse after ≥2 years during adjuvant ET.
• Progression within 1 year of completed adjuvant ET.
• Progression during ET used for >6 months in advanced disease, or progression within 1 month of completion of ET due to any cause.

For the treatment of HER2-positive and HR-positive advanced breast cancers, concomitant administration of a hormone inhibitor and an anti-HER2 agent is recommended after completion of chemotherapy. If chemotherapy cannot be administered, the first choice for metastatic disease is NSAI + trastuzumab (101), although recently the addition of pertuzumab is also recommended (II.B), and the combination of letrozole + lapatinib (102, 103) is approved for treatment in postmenopause.

### Chemotherapy in Metastatic Breast Cancer

#### General Considerations

• In the presence of a rapidly progressing tumour causing significant symptoms (visceral crisis) administration of combined chemotherapy should be considered, otherwise sequentially administered monotherapies are recommended, as these haven similar survival results and significantly lower toxicity (I.A).
• In metastatic disease, anthracyclines and taxanes are the most effective chemotherapy agents and are therefore recommended if they have not been used before. Reinduction of these agents should be considered if at least 1 year has elapsed since perioperative treatment (in the case of anthracyclines, the cumulative dose must be considered) (I.B).
• Additional recommended medicinal products: cyclophosphamide, capecitabine, vinorelbine, gemcitabine, carboplatin, sacituzumab govitecan or possibly cisplatin, and eribulin (see the recommended chemotherapy combinations) (I.A)(3).
• The duration of the recommended combination treatments is not defined: this depends on such factors as treatment efficacy, side-effects, and patient’s preferences. In general, therapy should be continued until progression or intolerable side-effects. The judgement of the patient must be included when defining tolerability (I.B) (2).
• Metronomic treatment refers to daily low-dose oral administration [cyclophosphamide + methotrexate (104), capecitabine + vinorelbine (105), capecitabine monotherapy (106)]. It is primarily recommended for the treatment of tumours with less aggressive biological characteristics. No comparison with standard treatments was performed (I.B).
• Addition of bevacizumab to first line therapy extends PFS but has no effect on overall survival. There is no known biomarker. Only selected patients can receive it as first-line treatment, in cases where other targeted treatments are not available (e.g., PD-L1 negative triple-negative cancer) and potential side-effects must also be considered. The best results were obtained with paclitaxel (107) (I.C).
• Based on the available results of a Phase 3 trial, the administration of platinum derivatives are primarily recommended for patients with a detectable germline BRCA pathological mutation (108) (I.B). Platinum-containing treatment should be considered for patients with a known BRCA mutation (II.B).
• The combination of CT with immunotherapy is an option in PD-L1 positive triple-negative breast cancer (see later).

#### Recommendations

• In the presence of a rapidly progressing tumour causing significant symptoms (visceral crisis) administration of combined chemotherapy should be considered, otherwise sequentially administered monotherapies are recommended (I.A).
• Anthracyclines and taxanes are the most effective chemotherapy agents and are therefore recommended if they have not been used before. Additional recommended medicinal products: cyclophosphamide, capecitabine, vinorelbine, gemcitabine, carboplatin, sacituzumab govitecan or possibly cisplatin, and eribulin.
• Metronomic treatment is primarily recommended for the treatment of tumours with less aggressive biological characteristics (II.B).
• Addition of bevacizumab to first line therapy extends PFS but has no effect on overall survival (I.B).
• The administration of platinum derivatives are primarily recommended for patients with a detectable germline BRCA pathological mutation (II.B).

### Systemic Treatment of HER2-Positive Advanced Breast Cancer (Locally Advanced, Stage IV or Recurrent Breast Cancer)

• For patients with HER2-positive recurrent/stage IV breast cancer anti-HER2 targeted therapy in combination with chemotherapy should be offered as first-line therapy in the absence of contraindications (I.A).
• Patients progressing on a HER2-targeted therapy should be offered additional subsequent treatment with a HER2-targeted therapy since continuous suppression of the HER2 pathway is beneficial (I.A).
• The choice of the HER2-targeted therapy will depend on previously administered therapy, relapse-free interval, patients’ preference and country-specific availability (V.C).
• The optimal sequence of available HER2-targeted therapies for recurrent/stage IV is currently not defined and relies on clinical experience (V.C).
• The optimal duration of HER2-targeted therapies is currently not known. The HER2-targeted therapy should be continued until progression or unacceptable toxicity (V.C).
• In patients achieving a complete remission, the optimal duration of maintaining HER2-targeted therapy is not known. Continuing HER2-targeted therapy until progression or unacceptable toxicity is recommended. Stopping HER2-targeted therapy after several years of sustained complete remission may be considered in some patients, particularly if treatment rechallenge is available in case of progression (V.C).
• For patients with HER2-positive/HR-positive recurrent or stage IV breast cancer, for whom the combination of HER2-targeted therapy and chemotherapy was given as first-line therapy, it is reasonable to use endocrine therapy in combination with HER2-targeted therapy as maintenance therapy after stopping chemotherapy, although this strategy currently has no supporting data from randomized clinical trials (V.C).
• HER2-targeted therapy and anthracyclines should not be given concurrently outside of a clinical trial (V.D).

### First-Line Therapy

• The preferred first-line option is pertuzumab plus trastuzumab in combination with chemotherapy. The combination of dual HER2-targeted and chemotherapy has proven to be more effective than the trastuzumab plus chemotherapy in terms of overall survival in this population. The preferred chemotherapy is docetaxel (I.A), or paclitaxel (I.B) (109, 110). Following the induction period with chemotherapy (at least six cycles/18 weeks of treatment), a maintenance therapy is recommended with the continuation of dual HER2-blockade, and the addition of endocrine treatment if ER-positive (II.A).
• For patients previously treated in the neo- and adjuvant setting with anti-HER2 therapy, the combination of chemotherapy and dual HER2-targeted therapy (trastuzumab plus pertuzumab) is an important option for first line therapy. (I.A) However, in the Cleopatra trial neo- and adjuvant trastuzumab was administered in only 10% of the patients and all of these had a trastuzumab free interval for more than 12 months (4).
• Currently there are no evidence supporting the continuation of dual HER2 blockade with trastuzumab and pertuzumab beyond progression, with the switch of the chemotherapy agent after progression. Therefore, dual HER2 blockade should not be given beyond progression (4) (V.C).
• When pertuzumab is not available, first-line regimens can include trastuzumab combined with a taxane or vinorelbine (111). Other alternative options are the combinations of trastuzumab and chemotherapy [such as trastuzumab + paclitaxel + carboplatin (109, 112), trastuzumab + capecitabine (113, 114). (III.C).
• First line options for HER2-positive/HR-positive disease include treatment with a HER2-targeted therapy plus chemotherapy or endocrine therapy. Chemotherapy combined with HER2-targeted therapy is still the optimal regimen for HER2+/HR + recurrent or stage IV cancer. However, endocrine therapy in combination with HER2-targeted therapy, such as trastuzumab and pertuzumab in combination with endocrine therapy is being a less toxic approach compared with HER2-targeted therapy plus chemotherapy (115, 116) (II.A). The approach of endocrine therapy plus a HER2-targeted agent should be reserved for highly selected patients, including those with contraindications to chemotherapy, patients with a strong preference against chemotherapy or those with a long disease-free interval (DFI), minimal disease burden and/or strong ER/progesterone receptor (PgR) expression (4). Combination regimens of HER2-targeted therapy plus endocrine therapy include the combination of an aromatase inhibitor ± trastuzumab, aromatase inhibitor ± lapatinib, or aromatase inhibitor + lapatinib + trastuzumab (117, 118).
• In case of patients progressing after the completion of adjuvant trastuzumab or trastuzumab plus pertuzumab therapy for early-stage disease, the choice of therapy depends on the interval that has elapsed since the completion of the HER2 inhibitor therapy. For patients with a DFI of 6–12 months after the completion of the adjuvant or neoadjuvant therapy trastuzumab and pertuzumab in combination with chemotherapy can be recommended. (II.B) For patient with an interval of less than 6 months between completion of the adjuvant or neoadjuvant therapy and the diagnosis of metastatic breast cancer T-DM1 therapy is the preferred first-line systemic therapy. (I.A) A combination of lapatinib + capecitabine is also an option (4, 119, 120) (I.C).

### Second-Line Trastuzumab-Based Therapy

• T-DM1 should be preferred in patients who have progressed after first-line, trastuzumab-based therapy. T-DM1 as a second-line therapy has been proven to be more effective compared to lapatinib + capecitabine, and provides an overall survival (OS) benefit (121). According to the results of DESTINY-Breast03 study trastuzumab-deruxtecan is more efficient than T-DM1 in second line setting and it may the preferred second line option in the future (122) (I.A).

### Third-Line and Beyond Therapy

• The therapeutic options for later lines of therapy depend on the patient’s preferences, toxicities developed during earlier therapies, and the availability of therapeutic agents.
• For patients who have progressed on T-DM1 or trastuzumab-containing therapy, the combination of trastuzumab plus lapatinib or lapatinib plus capecitabine is a reasonable option (119, 120, 123-127). (I.C) Only limited clinical data are available on the use of the combination after pertuzumab or T-DM1.
• Tucatinib plus trastuzumab in combination with capecitabine showed benefits in terms of median PFS and OS in comparison with trastuzumab plus capecitabine in patients who have progressed on trastuzumab, pertuzumab, and TDM1 including patients with brain metastases (128, 129). Tucatinib plus trastuzumab and capecitabine could be considered in third- and later line of therapy (I.A).
• Trastuzumab deruxtecan showed effectiveness in heavily pretreated patients with HER2-positive advanced breast cancer, and is a valid option in this setting (130) (III.A).
• The combination of neratinib plus capecitabine was compared with lapatinib plus capecitabine as third line or beyond therapy, showing a minimal benefit in PFS, and with no significant difference in OS (131) (I.C).
• Margetuximab plus chemotherapy showed only a small PFS benefit of 1 month when compared with trastuzumab plus chemotherapy in patients pretreated with trastuzumab, pertuzumab and T-DM1 (132) (I.B).
• Currently the new HER2 targeted agents, such as tucatinib, trastuzumab deruxtecan, neratinib, and margetuximab should be recommended for third and later lines of therapy.
• For later lines of therapy trastuzumab can be administered with several chemotherapeutic agents, such as: taxanes, vinorelbine, capecitabine, platinums, and eribulin (III.A).

#### Recommendation

• For patients with HER2-positive reccurent/stage IV breast cancer anti-HER2 targeted therapy in combination with chemotherapy should be offered as first-line therapy in the absence of contraindications (I.A).
• Patients progressing on a HER2-targeted therapy should be offered additional subsequent treatment and the HER2-targeted therapy should be continued until progression or unacceptable toxicity (I.A).
• It is reasonable to use endocrine therapy in combination with HER2-targeted therapy as maintenance therapy after stopping chemotherapy (III.B).
• HER2-targeted therapy and anthracyclines should not be given concurrently.
• The preferred first-line option is pertuzumab plus trastuzumab in combination with chemotherapy (I.A).
• Dual HER2 blockade should not be given beyond progression.
• When pertuzumab is not available, first-line regimens can include trastuzumab combined with a taxane or vinorelbine (I.A).
• The approach of endocrine therapy plus a HER2-targeted agent should be reserved for highly selected patients.
• T-DM1 should be preferred in patients who have progressed after first-line, trastuzumab-based therapy (I.A).
• Possible treatments for patients who have progressed on T-DM1 or trastuzumab-containing therapy: combination of trastuzumab plus lapatinib or chemotherapy not used before, lapatinib plus capecitabine, tucatinib in combination with trastuzumab and capecitabine, trastuzumab deruxtecan.

### Immunotherapy in Metastatic Breast Cancer

• Breast cancers are not among the highly immunogenic tumours.
• PD-1/PD-L1 inhibitors administered as monotherapy have demonstrated low efficacy.
• Combinations of these agents with chemotherapy have been studied in Phase 3 clinical trials.
  ○ In the KEYNOTE-119 trial, second or third line pembrolizumab given to patients with TN metastatic breast cancer did not provide overall survival benefit compared to chemotherapy given as monotherapy (133).
  ○ In the IMpassion130 trial (134), TN patients were treated with first-line nab-paclitaxel plus atezolizumab or placebo. The significant PFS benefit that was detected did not translate into OS benefit in the entire study population (135) (II.A).
  ○ Impassion131 trial failed to demonstrate any advantage (nor PFS or OS) in TN metastatic breast cancer when atezolizumab was added to paclitaxel (136) (II.A).
  ○ In the KEYNOTE-355 trial pembrolizumab added to chemotherapy (taxane or gemcitabine-carboplatin) the OS gain was clinically meaningful and statistically significant in PD-L1 positive tumours (CPS ≥ 10) (137) (I.A).
• Based on the results, determination of PD-L1 status is recommended for patients with irresectable, locally advanced, or metastatic TN breast cancer. A combination of pembrolizumab plus chemotherapy (taxane or gemcitabine-carboplatin) with CPS ≥ 10 or atezolizumab plus nab-paclitaxel with PD-L1 expression ≥1% is recommended as first-line therapy for these patients.

#### Recommendation

• Determination of PD-L1 status is recommended for patients with irresectable, locally advanced, or metastatic TN breast cancer.
• For patients with PD-L1 expression and no previous chemotherapy for their metastatic disease, a combination of atezolizumab and nab-paclitaxel (PD-L1 IC ≥ 1%) or pembrolizumab added to chemotherapy (taxane or gemcitabine-carboplatin, PD-L1 CPS ≥10%) is recommended (I.A).

## Supportive and Palliative Therapy

### Pharmacological Management of Bone Metastases

Among the metastases that emerge during the progression of breast cancer, bone metastases—detected in more than half of all cases—are the most common. Bisphosphonate therapy is the essential palliative treatment used in such cases.

• Administration of bisphosphonates (pamidronate, clodronate, zoledronate, or ibandronate) is recommended for bone metastases if,
  ○ The patient’s life expectancy is at least 3 months and the renal function is acceptable (creatinine clearance ≥30 ml/min). During bisphosphonate therapy, the patient’s renal function must be checked with the frequency defined in the SmPC (zoledronic acid: before each treatment, ibandronic acid: every 3 months) The current dose must be chosen in line with current renal function values.
  ○ Periodic measurement of electrolyte levels (calcium, magnesium, and phosphorus) is recommended, in parallel with imaging studies.
  ○ Appropriate vitamin D3 (400 IU/day) and calcium (500 mg/day) supplementation is required to prevent hypocalcaemia. The best option is to rely on albumin corrected calcium values.
  ○ Compared to pamidronate, zoledronic acid decreases the risk of sequelae of skeletal system events (pathological fracture, transection, necessity of bone irradiation or surgery) by 20% and its short infusion period is much more comfortable for patients.
  ○ Oral ibandronate is less effective in decreasing the risk of skeletal-related events but induces a marked lengthening of the interval before the first skeletal-related event.
  ○ Zoledronic acid therapy administered every 3 months^§^ can be considered the equivalent of the standard monthly therapy (I.B). An optional regimen is the administration of intravenous bisphosphonate therapy once a month at the beginning, and later the frequency can be reduced to once every 3 months (V.C).
• An additional therapeutic option for bone metastases is inhibition of the RANK (receptor activator of nuclear kappa-B) ligand.
  ○ Denosumab has been found to be more effective in the prevention of skeletal-related events than zoledronic acid.
  ○ Compared to bisphosphonates its administration (in the form of subcutaneous injection) is more comfortable for the patient, especially if the patient is not receiving other intravenous agents.
  ○ Denosumab can be used for the treatment of patients with severe renal impairment. The risk of hypocalcaemia is elevated under these conditions.
• The recommendation is to begin treatment with zoledronic acid or denosumab regardless of whether symptoms are present. Both medications are suitable for combinations with other anti-tumour medicines. In many countries, denosumab is approved for the treatment of progression detected during bisphosphonate therapy. However, denosumab was statistically superior to zoledronate in delaying both the first and subsequent skeletal related events and delayed worsening of bone pain (138).
• While the summary of product characteristics specifies that zoledronic acid (ibandronic acid, pamidronate, etc.) dosage should be adjusted according to the creatinine clearance values, the rules applied to denosumab therapy are less strict. A worsening of renal impairment is accompanied by an increase in the risk of hypocalcaemia as well as an increase in parathyroid hormone levels. It is therefore especially important to check the calcium levels of these patients regularly.
• The physician must consider the fact that regular bisphosphonate treatment is accompanied by a risk of developing osteonecrosis of the jaw. Bisphosphonate and denosumab therapy should be preceded by a dental consultation, and any intervention affecting the jaw as well as oral hygiene treatments such as dental cleaning, restorations, and treatment of mucosal inflammation. Appropriate vitamin D and calcium supplementation are also recommended to prevent hypocalcaemia (see above).
• In the presence of malignant hypercalcaemia, intravenous administration of bisphosphonate therapy is the proper approach.

Among adjuvant therapies affecting bone metabolism, the adjuvant use of bisphosphonates^§^ in patients in postmenopause improves disease-free survival and metastasis-free survival, and decreases the incidence of bone metastases and overall survival (139) (I.A). This effect is not seen in premenopause but it is present in both the HR-positive and HR-negative patient groups, i.e., it is not exclusively connected with endocrine therapy. A similar effect on survival could not be confirmed in the case of denosumab in early breast cancer, therefore this medication is not recommended associated with endocrine therapy (140, 141).

• In the presence of low oestrogen levels, breast cancer therapy recommended to be supplemented with bisphosphonates (e.g., zoledronic acid, oral clodronate or ibandronic acid every 6 months) (142), especially in high-risk disease (I.A)^§^. It is also recommended if the patient develops osteoporosis (I.A).
• During endocrine therapy, regular bone density measurements and, depending on the results, substitution are recommended in women receiving either an AI or OFS and men on ADT for >6 months with either a BMD T score of <2 or with two risk factors for fracture (I.A).
• Denosumab 60 mg every 6 months is the treatment of choice to prevent fractures in men on ADT and postmenopausal women with early breast cancer at low risk for disease recurrence (I.B).
• Weight-bearing exercise, smoking cessation, reduced alcohol intake and vitamin D supplements (and calcium) should be encouraged (I.B) (138).

#### Recommendation

• Administration of bisphosphonates (pamidronate, clodronate, zoledronate, or ibandronate) or denosumab is recommended for bone metastases and for malignant hypercalcemia (I.A).
• Periodic measurement of electrolyte levels (calcium, magnesium, and phosphorus) is recommended, in parallel with imaging studies.
• In the adjuvant setting and in presence of low oestrogen levels, breast cancer therapy recommended to be supplemented with bisphosphonates, especially in high-risk disease (I.A).

## Systemic Treatment of Special Subgroups

### Systemic Treatment of Hereditary Breast Cancer

In hereditary breast cancer based on germ cell mutation of the BRCA gene, the accepted guidelines for adjuvant systemic therapy are essentially identical to those applicable to non-hereditary (sporadic) breast cancers of identical immunophenotype, while there are several additional BRCA-specific treatment options for metastatic cases. Most breast cancers caused by germ cell BRCA (most frequently BRCA1) mutation are triple-negative. Based on gene expression studies, these are basal type cancers but likely represent a separate group. BRCA2-mutant tumours are typically luminal B-like. The genome of BRCA-associated tumours becomes unstable due to tumour suppressor gene errors and deficient DNA-repair mechanisms.

• Tumours originating from these errors are more sensitive to DNA-damaging cytostatic agents, mainly to those that lead to DNA cross-linking, such as platinum derivatives.
• The activity of platinum derivatives has been proven in both retrospective and randomized neoadjuvant trials. According to the results from multiple trials, pathological complete response of above 60% could be verified (143).
• The use of PARP inhibitors (olaparib, talazoparib) developed to inhibit PARP repair mechanisms provides PFS benefit compared to standard chemotherapy (144) (I.B).
• In the OlimpiA phase 3 adjuvant trial 1 year of olaparib added to standard adjuvant therapy in patients with gBRCA mutation and high-risk tumour (those with no pCR and CPS + EG score of ≥3) yielded a significant improvement in iDFS and DDFS. OS data are immature (145).

#### Recommendation

• Adjuvant/neoadjuvant treatment of patients is based on risk stratification. Accordingly, in cases of moderate-to high-risk triple-negative breast cancer the standard recommended systemic adjuvant treatment is the anthracycline-taxane sequence (AC-docetaxel or AC-paclitaxel); adjuvant platinum-containing therapy cannot be recommended due to a lack of sufficiently significant prospective data. Consider 1-year olaparib after standard (neo)adjuvant chemotherapy in high-risk patients.
• Similar principles are followed in metastatic cases, but also considering the type of previous adjuvant/neoadjuvant treatment, the interval since recurrence, the presence or absence of visceral crisis, etc. Platinum-based products, anthracyclines, taxanes and methylating agents are beneficial chemotherapies for HR-negative and HR-positive cases alike. The standard endocrine therapy algorithm can be used in hormone-sensitive cases. These treatment options can be supplemented with PARP inhibitors suitable for both HR-negative and HR-positive subgroups (143, 146-148). The optimal order of the sequence has not yet been established, but it is known that their efficacy is decreased by recent prior platinum therapy or clear resistance to platinum-containing agents. The latest ESO-ESMO international consensus provides level I. c evidence for the recommendation of platinum-based therapy for metastatic BRCA-associated triple-negative or hormone-resistant breast cancer if the patient has received prior anthracycline (with or without taxane), or PARP-inhibitor, or if the patient has received prior anthracycline (with or without taxane), and endocrine therapy for the luminal type.

For both early and advanced disease participation in clinical trial is recommended.

### Systemic Treatment of Male Breast Cancer

Male breast cancer is a rare disease; its incidence is 1:100 that of female breast cancer. Due to the low incidence of the disease, there are no separate therapeutic recommendations, therefore it is assumed that—since generally male patients could not be included in trials—the principles of treatment are like those used for female patients. However, increasing amounts of data support the hypothesis that male breast cancer and female breast cancer are separate diseases (149-156).

The incidence of the BRCA1 mutation is 1%–5% among male patients, and of the BRCA2 mutations 5%–10%, and the presence of the mutation indicates worse prognosis (149). It is important to bear in mind during follow-up that the BRCA mutation makes the patient susceptible to prostate cancer and pancreatic cancer.

#### Recommendation

• During systemic therapy for early-stage cancer (neo)adjuvant chemotherapy should be chosen according to the treatment principles used for female breast cancer. For HER2-positive tumours 1 year of trastuzumab therapy is recommended, just like for women (IV).
• Most male breast cancers are hormone-sensitive, and therefore endocrine therapy is one of the essential parts of adjuvant treatment. The standard therapy is still 5 years of adjuvant tamoxifen, but based on positive data from the ATLAS trial (157) 5 + 5 years extension can be used in high-risk cases. In addition to tamoxifen therapy, it is very important to support adequate compliance, because according to data in the published literature compliance is lower among male patients, which can potentially lead to treatment failure.
• The preferred endocrine agent is tamoxifen. Use of an aromatase inhibitor can be considered if the patient is unable to tolerate tamoxifen, but it must be combined with an LHRH analogue.
• The principles governing systemic therapy of advanced metastatic disease are identical for men and women.
  ○ If the tumour is hormone-sensitive, endocrine therapy is the first choice, and an additional endocrine therapy sequence can be administered in case of progression. However, the evidence for these treatments is significantly weaker than it is for women.
    – Traditionally the standard first-choice medicine is tamoxifen, with an expected response rate of over 80%.
    – If there is disease progression during tamoxifen treatment or if tamoxifen is contraindicated for a different reason, then endocrine therapies used for the treatment of female breast cancer are administered, and aromatase inhibitors are to be combined with an LHRH analogue (or orchiectomy).
    – The most recent (2019) guidelines from the ABC5 consensus conference (4) included the recommendation of standard therapy consisting of endocrine therapy combined with CDK4/6 inhibitor (aromatase inhibitor or fulvestrant) as first or second-line therapy for male metastatic breast cancer, in the absence of visceral crisis, and in combination with LHRH analogue.^§^ This is currently considered an off-label treatment in many countries and requires approval from both the national drug and health care and financing authorities.
    – If there are ongoing clinical trials, male patients should be encouraged to join.
    – Endocrine therapy should be continued until the tumour becomes endocrine resistant or visceral crises develop.
    – Second- and third-line treatments after CDK 4/6 inhibitor combination can be similar as in women (extrapolation of results) or reinduction of tamoxifen is possible, depending on previous treatments.
    – Options for endocrine therapy in later lines include aminoglutethimide, androgens, corticosteroids, and LHRH analogue therapy.
  ○ As in female breast cancer, the presence of visceral crisis or endocrine resistance of HR-negative disease necessitates the use of chemotherapy. The treatment principles are identical for female and male breast cancers.
  ○ Similarly, the recommendation for HER2-positive breast cancer is the use of HER2 inhibitor combination according to the same principles as for female patients.
  ○ There are very limited clinical data on the use of mTOR and PARP inhibitors, but the principles are identical to those applied for female patients^§^ (V.C) (156).

### Systemic Treatment of Occult Breast Cancer

Occult breast cancer may present in the form of axillary lymph node metastasis (this is the most common form) or in rare cases in the form of other distant metastases (in abdominal organs and lymph nodes, omental infiltration, etc.). The latter cases are more characteristic of lobular breast cancer, and, like axillary metastatic carcinoma of undetectable breast cancer, the primary tumour cannot be detected in the breasts even with detailed examinations. The recommended treatment for these cases is metastatic protocol based on immunophenotype.

#### Recommendation

• The treatment of cases manifesting as isolated axillary lymph node metastases is identical to that of locoregional or locoregionally advanced disease.
• Endocrine therapy is recommended in the presence of high ER/PR levels and low proliferation rate.
• Chemotherapy is recommended in the case of visceral crisis.
• Verified HER2 positivity requires HER2 inhibitor treatment using the same rules as described above.

### Breast Cancer During Pregnancy and Lactation

Malignant diseases diagnosed during pregnancy are rare, accounting for approximately 0.02%–0.1% of all pregnancies. Nevertheless, as women increasingly delay pregnancy the incidence of malignant tumours is expected to increase. Breast cancer during pregnancy accounts for 3% of all cases, amounting to approximately one case per 3,000 pregnancies.

#### Diagnosis

Due to physiological hyperplasia during pregnancy, the breast parenchyma becomes more solid and nodular on palpation, making physical examination more difficult. Tumours are typically diagnosed in the form of palpable nodules with several months’ delay. The pregnancy-associated breast cancer is considered to have a poorer prognosis even when controlled for stage and hormone receptor status (158).

Ultrasonography is the primary tool for the evaluation of breast complaints in pregnant women. If necessary (e.g., suspected tumour, DCIS/EIC component, etc.) mammography can be performed, observing radiation protection guidelines. Breast MRI is more difficult due to the necessity for contrast material, as well as the increased abdominal circumference and prone position during the scan. Generally, the administration of MRI contrast medium during pregnancy is a relative contraindication, can be applied “if the clinical status of the woman necessitates it” (the label of contrast media should be checked before use). There are major differences between contrast media and their uses in different countries, therefore the summary of product characteristics should be followed in all cases. Ultrasound is a safe method for staging and shielded X-ray may be used. CT scan and bone scintigraphy are contraindicated.

To determine the pathological diagnosis a core biopsy should be performed, since its sensitivity is approximately 90%. In all cases the pathologist must be informed of the patient’s pregnancy.

#### Treatment

The treatment should be based on the disease stage (159).

• Breast cancer during pregnancy requires continuous monitoring of the patient by the gynaecologist. Pre-term delivery should be avoided if possible.
• Systemic treatment:
  ○ Breast cancer therapy can be administered during pregnancy, after first trimester, and termination of the pregnancy by itself does not improve the prognosis.
  ○ During the first trimester chemotherapy is contraindicated.
  ○ During the second and third trimesters chemotherapy can be administered.
  ○ Starting at the second trimester all chemotherapy treatments must be preceded by antenatal consultation and fetal monitoring is recommended.
  ○ After week 35 (or at least 3 weeks before the expected delivery date) chemotherapy is not recommended to avoid delivery complications caused by pancytopenia.
  ○ The largest body of experience is with the combination of doxorubicin/epirubicin and cyclophosphamide.
  ○ There is less experience with the use of taxanes; if clinically indicated, a weekly regimen of paclitaxel can be recommended during the second and third trimesters.
  ○ Anti-HER2 therapy and hormone therapy are contraindicated during pregnancy.
  ○ The response to chemotherapy must be quantified, and the condition of the fetus must be monitored regularly before each anthracycline-containing treatment or every 3–5 weeks when administering local taxane treatment.
  ○ Monitoring of tumour response is performed according to daily routine after 3–4 cycles of anthracycline or 12 weeks of taxane therapy, but control studies can be performed at shorter intervals if clinically justified.
  ○ If no tumour response is detected, the oncology team should revise its opinion accordingly. Results from the patient’s antenatal monitoring should be present to determine further actions.
  ○ Breastfeeding is not recommended during chemotherapy.
  ○ Safe anti-emetic agents include ondansetron, lorazepam and dexamethasone.
  ○ Corticosteroids are not contraindicated, prednisolone may be safer in the first and second trimester (160).
  ○ G-CSF can be safely used.
• Surgery can be performed in any trimester. According to American recommendations breast surgery can be performed after week 25, but an obstetrician and a perinatal specialist must be available and ready to intervene in case of early delivery (161).
  ○ It should be taken into consideration for choosing the type of surgery (mastectomy or breast-conserving operation) that radiotherapy is possible only after delivery. If radiation therapy can be delayed until after the delivery, breast-conserving surgery is not inferior to mastectomy.
  ○ Appropriate axillary staging is a required part of surgery. If the axilla is clinically negative, sentinel lymph node biopsy can be performed.
  ○ According to the latest recommendations, primary reconstruction is accepted after mastectomy.
• Radiation therapy is contraindicated during pregnancy.

### Treatment of Phyllodes Tumour

• The primary treatment is surgery.
• Neither adjuvant endocrine therapy nor adjuvant chemotherapy provide any confirmed benefits. In rare cases of systemic progression, systemic treatment according to the soft tissue sarcoma protocol is recommended.

## Discussion

This recommendation reflects the content of the main international guidelines. However, there are minor differences between these guidelines, and the members of the board have chosen the one that best suits their regional circumstances. It may deviate from one or other of the international guidelines in this regard and may give rise to further discussions. The manuscript was completed in February 2022 and does not contain the scientific results published thereafter. The authors are convinced that the discussion leading to the drafting of this manuscript and the guideline itself represent a significant advance in the care of breast cancer patients in the Central and Eastern European region.

This is part 2 of a series of 6 publications on the 1st Central-Eastern European Professional Consensus Statements on Breast Cancer covering imaging diagnosis and screening, pathological diagnosis, surgical treatment, systemic treatment (present paper), radiotherapy of the disease and related follow-up, rehabilitation and psycho-oncological issues.

## Footnotes

1 Urokinase plasminogen activator/plasminogen activator-inhibitor-1 (uPA/PAI-1) is also an option, used in some countries).

2 When IHC is used for classification, it is recommended to apply the “-like” suffix in order to differentiate genome- and IHC-based definitions (e.g., luminal A-like).

3 According to the health care financing rules currently in force, after adjuvant therapy with trastuzumab the administration of the same agent in metastatic cancer requires measurement of HER2 overexpression, while measurement of HER2 overexpression must be carried out for lapatinib therapy.

## Author’s Note

The consensus document contains product placement without the intention of advertising. Each complex molecular test is unique, and although these can be described without indicating their name (for example with the number of genes tested), not everyone will necessarily understand what this refers to. For this reason, and adopting the practice used in some of the source works, the tests are listed under their trade name.
