# Corrigendum: Systemic treatment of breast cancer. 1st Central-Eastern European professional Consensus Statement on breast cancer

**DOI:** 10.3389/pore.2023.1610954

**Published:** 2023-01-31

**Authors:** Gábor Rubovszky, Judit Kocsis, Katalin Boér, Nataliya Chilingirova, Magdolna Dank, Zsuzsanna Kahán, Dilyara Kaidarova, Erika Kövér, Bibiana Vertáková Krakovská, Károly Máhr, Bela Mriňáková, Béla Pikó, Ivana Božović-Spasojević, Zsolt Horváth

**Affiliations:** ^1^ Department of Clinical Pharmacology, National Institute of Oncology, Chest and Abdominal Tumours Chemotherapy “B”, Budapest, Hungary; ^2^ Center of Oncoradiology, Bács-Kiskun County Teaching Hospital, Kecskemét, Hungary; ^3^ Department of Oncology, Szent Margit Hospital, Budapest, Hungary; ^4^ Clinic Center of Excellence, Heart and Brain Hospital, Science and Research Institute, Medical University-Pleven, Pleven, Bulgaria; ^5^ Oncology Centre, Semmelweis University, Budapest, Hungary; ^6^ Oncotherapy Clinic, University of Szeged, Szeged, Hungary; ^7^ Kazakh Institute of Oncology and Radiology, Almaty, Kazakhstan; ^8^ Institute of Oncotherapy, Faculty of Medicine, University of Pécs, Pécs, Hungary; ^9^ 1st Department of Oncology, Faculty of Medicine, Comenius University, Bratislava, Slovakia; ^10^ Medical Oncology Department, St. Elisabeth Cancer Institute, Bratislava, Slovakia; ^11^ Department of Oncology, Szent Rafael Hospital of Zala County, Zalaegerszeg, Hungary; ^12^ County Oncology Centre, Pándy Kálmán Hospital of Békés County Council, Gyula, Hungary; ^13^ Institute for Oncology and Radiology of Serbia, Daily Chemotherapy Hospital, Belgrade, Serbia

**Keywords:** early breast cancer, locally advanced breast cancer, adjuvant treatment, neoadjuvant treatment, metastatic breast cancer, inflammatory breast cancer, guideline

In the original article, the reference for **118** was incorrectly written as:

“Johnston SRD, Hegg R, Im SA, Park IH, Burdaeva O, Kurteva G, et al. Phase III, Randomized Study of Dual Human Epidermal Growth Factor Receptor 2 (HER2) Blockade With Lapatinib Plus Trastuzumab in Combination With an Aromatase Inhibitor in Postmenopausal Women With HER2-Positive, Hormone Receptor-Positive Metastatic Breast Cancer: ALTERNATIVE. Journal of clinical oncology: official journal of the American Society of Clinical Oncology. 2018;36(8):741-8.”

It should be

“Johnston SRD, Hegg R, Im SA, Park IH, Burdaeva O, Kurteva G, et al. Phase III, Randomized Study of Dual Human Epidermal Growth Factor Receptor 2 (HER2) Blockade With Lapatinib Plus Trastuzumab in Combination With an Aromatase Inhibitor in Postmenopausal Women With HER2‐Positive, Hormone Receptor-Positive Metastatic Breast Cancer: Updated Results of ALTERNATIVE. J Clin Oncol. 2021;39(1):79–89. doi: 10.1200/JCO.20.01894.”

The authors apologize for this error and state that this does not change the scientific conclusions of the article in any way. The original article has been updated.

